# Editorial: The shape of lives to come

**DOI:** 10.3389/fpsyg.2023.1154577

**Published:** 2023-03-22

**Authors:** Michelle Maiese, Arran Gare, Julian Kiverstein, Joel Krueger, Robert Hanna

**Affiliations:** ^1^Department of Philosophy, Emmanuel College, Boston, MA, United States; ^2^Department of Philosophy and Cultural Inquiry, Swinburne University of Technology, Hawthorn, VIC, Australia; ^3^Department of Psychiatry, Amsterdam University Medical Center, Amsterdam, Netherlands; ^4^Department of Sociology, Philosophy, and Anthropology, University of Exeter, Exeter, United Kingdom; ^5^Independent Researcher, Boulder, CO, United States

**Keywords:** mind shaping, metaphor, cognition, embodiment, framing

The five articles under this Research Topic constructively and critically discuss various dimensions and implications of The Theory of Thought-Shapers (TTS) (Hanna and Paans, 2021), thereby collectively making a strong case for its cogency and truth. TTS says that thought-shapers—i.e., mental frames, especially including metaphors, analogies, images, schemata, stereotypes, symbols, and templates—partially causally determine, form, and normatively guide (i.e., shape) our essentially embodied human minds-&-lives. Such shaping can occur more negatively, by means of mechanical, constrictive thought-shapers, or more positively, by organic, generative thought-shapers. TTS, which is empirically testable, is embedded within (a) a fundamental metaphysics of the mind-body relation and mental causation, *the essential embodiment theory* (Hanna and Maiese, 2009), and (b) a general theory of how social institutions shape people's lives for worse or for better, *the mind-body politic* (Maiese and Hanna, 2019). Against that theoretical backdrop, these five articles begin to reveal both the potential dangers of thought-shaping, and how thought shaping might be implemented in a more constructive way.

Three of the articles in this Research Topic present contemporary case studies that reveal the centrality of affective thought-shaping. The other two examine how TTS can be extended into novel domains.

The first three case-studies outlined below deal, respectively, with (i) the COVID-19 pandemic insofar as it has applied to jobs, family support, and family life, (ii) the everyday lives of farmers in an urban wetland near Mexico City, Xochimilco, and (iii) parental or guardian hoping as it applies to families or other communities. All three studies examine how thought-shaping operates on people's desires, emotions, and feelings, and thereby influences their intentional actions. In “*COVID-19 in the United States as affective Frame*,” Protevi deploys the notion of an “affective frame”—i.e., the essentially embodied emotional scaffolding of a social situation—in order to work out a case study of a double-binding negative affective frame. This double-bind arises due a tension between, on the one hand, having a job as a care-giver who risks catching the disease, and on the other hand, being a household provider of income who needs to perform another kind of care-giving labor at home, while also risking passing on the disease to their family. Protevi's discussion highlights a collective of harmful social practices that impact workers who simultaneously strive to satisfy the demands associated with their roles as both breadwinners and caring professionals. In “*Sense of agency, affectivity and social-ecological degradation: An enactive and phenomenological approach*,” Siqueiros-García et al. examine the impact of environmental change and degradation on people's affective lives. Deploying the methods of phenomenology, enactivism, and ecological psychology, they argue that the loss of a traditional form of life in Xochimilco flows from the degradation of socio-ecological systems, which limits subjects' opportunities to relate to other people and the natural environment in a meaningful way. This loss of meaningful interactions with the environment, in turn, generates a feeling of loss of control, which affectively manifests among the farmers as anxiety, frustration, and negative stress. The destructive nature of this affective shaping thereby cripples their sense of agency. And in “‘*Bringing new life in': Hope as a know-how of not knowing*,” Cuffari et al. present another case study, this time the practice of guardian or parental hoping, which they describe as a form of know-how that shapes subjects' cognitive and affective processes. In their view, hope is not an individual emotion, but rather consists in a shared form of social activity. In particular, it involves linguistic activity and the navigation of uncertainty via the reframing of utterances. These authors also identify particular impediments to and facilitators of hope that function as restrictive or generative thought-shapers, respectively.

The two novel domains into which TTS is extended are (i) neuro-immunology and (ii) the larger context of human representational activities, including but not necessarily restricted to language. In “*Action-shapers and their neuro-immunological foundations*,” Paans and Ehlen extend the theory of thought-shapers (TTS) to what they call *action shaping*, by presenting a theory of how neuro-immunological processes affect our intentional abilities and our capacity to act. Two of the examples that they discuss are chronic stress and high levels of sugar intake, both of which shape people's capacity to form intentions and execute actions. And in “*Thought-shapers embedded*,” Kondor proposes that thought-shapers are built on a more basic and richer set of structures that consists of representational skills, tools, and social institutions, thereby *embedding* thought-shapers.

Taken together, these five articles testify to the theoretical power of TTS and its potential application to a wide range of different topics. Our hope is that they initiate new conversations about the complex interplay between social institutions and human minds-&-lives.

## Author contributions

RH wrote the initial draft of this editorial, which was then edited and revised in light of comments raised by MM, AG, JKi, and JKr. All authors contributed to the article and approved the submitted version.
